# Comparison and convergent validity of five Mediterranean dietary indexes applied to Brazilian adults and older adults: data from a population-based study (2015 ISA-Nutrition)

**DOI:** 10.1017/jns.2022.123

**Published:** 2023-01-26

**Authors:** Amália A. Bastos, Paula V. Félix, Michelle A. Castro, Regina M. Fisberg, Antônio A. M. Silva, Mary Yannakoulia, Sandra M. L. Ribeiro

**Affiliations:** 1 Department of Nutrition, Public Health School, University of Sao Paulo, São Paulo, Brazil; 2 School of Feeding Coordination, São Paulo City Hall, São Paulo, Brazil; 3 Department of Public Health, Federal University of Maranhão, São Luís, Brazil; 4 Department of Nutrition and Dietetics, School of Health Sciences and Education, Harokopio University, Athens, Greece; 5 School of Arts, Sciences and Humanities, University of Sao Paulo, São Paulo, Brazil

**Keywords:** Adherence, Confirmatory factor analysis, Dietary indexes, Mediterranean dietary indexes, Mediterranean dietary pattern

## Abstract

Different dietary indexes are proposed to investigate adherence to the Mediterranean diet (MD). However, they are based on different methodologies, and limited research has compared them to each other, particularly in non-Mediterranean populations. We aimed to compare five indexes intended to measure adherence to the MD. The sample was composed of adults and older adults (*n* 1187) from 2015 ISA-Nutrition, a cross-sectional population-based study in São Paulo, SP, Brazil. Dietary data obtained through two 24-h dietary recalls (24HDR) from which the Mediterranean diet scale (MDS), Mediterranean diet Score (MedDietscore), Mediterranean dietary pattern (MDP), Mediterranean Adequacy Index (MAI) and Mediterranean-Style Dietary Pattern Score (MSDPS) were calculated. The correlations and agreements between them were analysed by Spearman's correlation and linearly weighted Cohen's Kappa coefficients, respectively. Confirmatory factor analyses (CFAs) were applied to investigate their convergent validity. The highest correlations were found between MDP and MAI (*r* = 0⋅76; 95% CI 0⋅74–0⋅79) and between MDP and MDS (*r* = 0⋅72; 95% CI 0⋅69–0⋅75). The greatest agreements observed were moderate, between MDP *v*. MAI (*κ* = 0⋅57, *P* < 0⋅001) and MDP *v*. MDS (*κ* = 0⋅48, *P* < 0⋅001). The goodness-of-fit of CFA for MedDietscore (RMSEA = 0⋅033, 90% CI 0⋅02–0⋅042; SRMR = 0⋅042) and MSDPS (RMSEA = 0⋅028, 90% CI 0⋅019–0⋅037; SRMR = 0⋅031) had acceptable values for absolute fit indices. Vegetables, olive oil, MUFA:SFA ratio and cereals with legumes were more relevant to characterise the MD (factor loadings ≥0⋅50). The MDS, MAI and MDP classified the population similarly, but the MedDietscore showed better performances in evaluating adherence to the MD. These results provided guidance for the most appropriate Mediterranean dietary index to be applied in non-Mediterranean populations.

## Introduction

The Mediterranean diet (MD) is well known as a healthy and sustainable dietary pattern (DP) with an emphasis on fresh, locally produced foods, primarily plant-based, preferably minimally processed from their natural state^(1,2)^. Considering these relevant aspects, experts have discussed possibilities of adaptations and incorporation of its characteristics in non-Mediterranean countries, bringing a better quality of food habits in these regions^(1,3)^. To do so, the first step is to know the degree of adherence to this dietary pattern of a given population, which generally is performed from a hypothesis-driven (*a priori*) approach^(4)^. Although most of the indexes were developed and validated in Mediterranean populations, many studies have used them to evaluate the diet quality of non-Mediterranean populations^(5)^.

Relevant health benefits have been attributed to MD, and for this reason, available indexes have been used in different regions of the world, undergoing adaptation studies^(6)^. These adaptations revolve around the score's range, adherence indicators (nutrient, food or both), the scoring system and cut-off points^(6)^. It is important to note that these indexes present structural differences between them and that any choice for non-Mediterranean populations should be preceded by studies comparing the best performance of each one since diet quality indexes often lack sensitivity to culture.

An essential question in this regard is whether an index based on the dietary habits of the Mediterranean population can be applied to non-Mediterranean regions, considering that non-Mediterranean countries have their living and eating habits. Following these thoughts, in the USA, some MD indexes were developed or adapted to meet the particularities of that population^(7–10)^. At the same time, some traditional Mediterranean indexes non-adapted to the USA population, such as Mediterranean Diet Score (MedDietscore) and Mediterranean Diet scale (MDS), have also been used in studies with this population^(11–13)^. In addition, a recent systematic review and meta-analysis of observational studies investigated the impact of adherence to the Mediterranean diet on Metabolic Syndrome parameters. Overall, the studies in Sweden, Iran, Korea, Morocco, Australia and others adopted nine different indexes^(14)^. Therefore, in different countries, or even in a single country, a range of indexes has been adopted.

Considering the scarcity of investigations comparing the performance of these different indexes, this choice is commonly made without being based on previous studies. Also, investigations regarding their components, structure, the methods applied and their performance in assessing the adherence to this DP, i.e. whether the different indexes are measuring the same construct, are poorly accomplished. One important source of bias when adopting MD indexes not locally validated is the need to include non-Mediterranean foods commonly consumed in these countries. The existing MD indexes are generally food groups traditionally consumed by Mediterranean populations; other foods locally consumed may be inappropriately computed.

These gaps make it difficult to identify the most appropriate index employed in each population. Since no MD index is developed and adapted to the multiethnic Brazilian population, studies carried out with this group also adopt a convenient range of indexes. We hypothesise the existence of essential differences between the indexes commonly used to measure adherence to the MD. We hope to show results that can guide the choice of a more appropriate index in this population. Thus, the present study aims to computing scores, compare the performance and investigate the convergent validity of five Mediterranean dietary indexes, which means if these indexes converge to the same hypothesis, reaching the objective of identifying adherence to the MD using a population-based sample of adults (20–59 years) and older adults (age ≥60 years) living in São Paulo, SP, Brazil.

## Methods

### Population and study design

The present study includes data from the 2015 Health Survey of São Paulo with a focus on nutrition (2015 ISA-Nutrition), a cross-sectional population-based study. People residing in permanent private households in the urban area of São Paulo city were randomly selected from September 2014 to December 2015.

Details about the 2015 Health Survey of São Paulo (ISA-Capital) sampling process were published elsewhere^(15)^. In brief, the sample from São Paulo City, SP, Brazil, was stratified by clusters, performed in two stages: census tracts, stratified in five Regional Health Coordination of São Paulo City (north, mid-west, southeast, south and east) and domiciles. For statistical analysis, sampling survey weights were applied to account for the complex and stratified cluster sampling design. All the persons from the families who belonged to the demographic domain selected in the study and met the inclusion criteria (people of both genders, aged 12+ years and residing in the urban area of São Paulo during the survey period) were interviewed. The exclusion criteria were: individuals with chronic alcoholism and those on an enteral or parenteral diet.

To minimise the effects of losses and refusals, more extensive independent random selections were performed, securing a sample that allowed the estimation of proportions of the differences of 0⋅50. The sampling error was seven percentage points, considering a 95% confidence level and a delineation effect of 1⋅5.

Among the 4059 participants of the 2015 ISA-Capital, 1737 were randomly selected to participate in the first phase of 2015 ISA-Nutrition, a study that included diet and nutritional status data. More detailed information about 2015 ISA-Nutrition can be found elsewhere^(16)^.

The Research Ethics Committee of the School of Public Health of the University of São Paulo approved the 2015 ISA-Capital and 2015 ISA-Nutrition surveys (protocols 32344014.3.3001.0086 and 30848914.7.0000.5421, respectively). All individuals included in the survey had their data collected only after signing the Informed Consent Form.

Initially, the present study comprised adults (20–59 years) and older adults (age ≥60 years) of both genders who had their habitual intake obtained through two 24-h dietary recall (24HDR) in the 2015 ISA-Nutrition survey, totalling 1188 individuals. It was impossible to obtain the score of one of the indexes for one participant, resulting in his exclusion and thus, totalling 1187 participants in the present study. Supplementary Figure 1 brings the description of the sample in the 2015 Health Survey of São Paulo with Focus on Nutrition Study (2015 ISA-Nutrition) included in the present study.

### Diet assessment

Food consumption data were obtained from two non-consecutive 24HDR, applied for a 1-year interval, covering all seasons and days of the week. The first 24HDR was collected from 1188 participants during a first domicile visit and followed the Multiple-Pass Method (MPM)^(17)^ to reduce errors in dietary measurement^(18)^. The second 24HDR was collected from a subsample of 367 adults and older adults using the Automated Multiple-Pass Method (AMPM)^(17,19)^ through telephone calls using the interview system incorporated into the Nutrition Data System for Research (NDSR, version 2014, developed by the Nutrition Coordination Center, University of Minnesota, Minneapolis, USA), then entered into the NDSR software (version 2014). Subsequently, habitual dietary intake was estimated using the Multiple Source Method (MSM) programme^(20)^, a web-based tool accessed online at http://msm.dife.de/. This statistical method estimates the usual intake of food and nutrients consumed by the population reported in the two 24HDR^(20)^.

### Choice of the indexes of adherence to the Mediterranean diet

Firstly, we proceeded to an in-deep reading of two systematic reviews^(21,22)^, recent publications at the beginning of this study, aiming to identify the criteria for the choice of an index and to evaluate the conceptual, applicability and psychometric properties of MD indexes. One of them included only Mediterranean indexes, citing twenty-eight tools. While the second evaluated *a priori* dietary indexes in general, citing twenty-one Mediterranean dietary indexes. Together, these two systematic reviews encompassed twenty-four different indexes evaluating the MD. Secondly, we selected from these indexes, excluding the ones with the following aspects: (i) to be a variation of the MDS or MedDietScore; (ii) to have a structured questionnaire with closed-ended questions, making it impossible its use with data from the 24HDR method; (iii) have a metric in which consumption was not transformed into normalised scoring range. After applying these criteria, nineteen dietary indexes (Supplementary Table 1) were excluded^(7–9,23–37)^, and five were selected for the present study.

The five selected indexes were calculated and compared concerning their similarities and differences, as detailed below.

### Mediterranean diet scale (MDS)

The MDS was developed by Trichopoulou *et al.*^(38,39)^ using dietary data from the Greek population. Individuals whose consumptions of fish, vegetables, legumes, fruits and nuts and cereals, as well as the MUFA:SFA ratio, were below or above the median of consumption, received the score of ‘zero’ or ‘one’, respectively. An inverse score was assigned for meat and dairy, components presumed to be detrimental, which are rarely non-fat or low-fat. All food components consumed were adjusted for total energy intake for both sexes. For alcohol, a value of one was assigned to moderate consumption by men (which corresponds to an amount of 10 and 50 g/d) and by women (5 and 25 g/d) and zero for consumption below or above these values for each sex. Thus, the total MDS ranged from 0 to 9, with higher scores indicating greater adherence to the Mediterranean dietary pattern (MDP).

### Mediterranean Diet Score (MedDietscore)

The MedDietscore was proposed by Panagiotakos *et al.*^(40)^ based on Greek Mediterranean traditional dietary habits. The scoring is based on the consumption of eleven main dietary components of the Mediterranean diet (unrefined cereals, fruits, vegetables, potatoes, legumes, olive oil, fish, red meat, poultry, whole dairy products and alcohol) according to their position in the pyramid of the traditional Mediterranean Diet. Each component scores from ‘zero’ to ‘five’ points. For the typical food components of the Mediterranean diet (unrefined cereals, fruits, vegetables, potatoes, legumes, olive oil and fish), the intake recommendation is either daily or more than four servings per week. Therefore, a score of ‘zero’ is given for no consumption, a score of ‘one’ when the consumption ranges from 1 to 4 servings/month, a score ‘two’ to 5 to 8 servings/month and a score of ‘three’ to 9 to12 servings/month, a score ‘four’ to 13 to18 servings/month and a score ‘five’ for more than 18 servings/month. The inverse score was applied for consuming foods that differed from this dietary pattern (meat and its derivatives, poultry, and whole dairy products). Especially for alcoholic beverages, points ranging from ‘zero’ to ‘five’ are assigned according to the amount of consumption in ml per day. A score ‘five’ is assigned to consumption <300 ml/d, a score of ‘zero’ to no consumption or consumption >700 ml/d and a score ‘four’ to ‘one’ for consumption of 300–400 ml/d, 400–500 ml/d, 500–600 ml/d, 600–700 ml/d, respectively (assuming 100 ml at 12 g ethanol concentration). As each of the eleven dietary components of the index are scored from 0 to 5, there was no need for truncation for the total score. However, there was need for truncation of individual food component scores. The cut-off points (recommendations in servings per month) were truncated to integers in each 0⋅5. Thus, the total MedDietscore score ranges from 0 to 55, with higher scores indicating greater adherence to the MDP.

### Mediterranean Adequacy Index (MAI)

Constructed with data from the population of Italy, the MAI is based on the percentage of the total daily energy intake of four food groups: (i) carbohydrate group (bread, cereals, dried pulses, potatoes); (ii) protective group (vegetables, pulses, fresh, fruits, fish, alcoholic beverages such as red wine, vegetable oils); (iii) land animal group (milk, cheese, meat, eggs, animal fats and margarine) and (iv) sweets group (drinks, sweets, cakes, pies and cookies, sugar). The MAI score is obtained by dividing the sum of the percentage of the total daily energy intake of groups one and two by the sum of the total daily energy intake of groups three and four. Thus, its final score is interpreted as a ratio that can range from 0 to 100, with higher ratios meaning greater adherence to the MD.^(41)^

### Mediterranean-Style Dietary Pattern Score (MSDPS)

Developed using data from the population of North America, the MSDPS calculation includes thirteen components: whole grains, fruits, vegetables, wine, fish and seafood, olives, legumes and oilseeds, potatoes and other starchy roots, olive oil, chicken, red meat, sweets, eggs and dairy products. Each component (except olive oil) is scored from 0 to 10 according to the Mediterranean Diet Pyramid recommendation. One point is also deducted proportionally to the number of servings consumed above the recommended intake for the group. Negative scores default to zero. The olive oil score includes only 0, 5 or 10 points, equivalent to non-consumption of olive oil, use of olive oil and other vegetable oils, and exclusive use of olive oil, respectively.

Subsequently, the thirteen component scores are summed, and the total is standardised on a scale of 0–100. For this, the total score is divided by the maximum score of 130 points and multiplied by 100. The total standardised score is multiplied by a factor ranging from zero to one (reflecting 0–100% of energy intake attributed to foods that are part of the MDP). Thus, for each individual, the MSDPS was calculated by the following equation^(10)^:
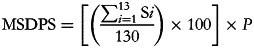
where S*i* is the individual item score; *P* is the proportion of total energy intake from Mediterranean diet pyramid foods.

### Mediterranean dietary pattern (MDP)

Developed based on the consumption of the population of Spain, MDP calculation is carried out considering the following steps: the daily energy consumption of cereals, fruits, vegetables, legumes, meat and dairy products; the adjusted energy intake of each of these foods is standardised to *z*-scores; before standardisation, centralised alcohol consumption for men (30 − |30 − absolute alcohol intake|) and for women (20 − |20 − total alcohol consumption|) to obtain the highest value for men consuming 30 g/d or women consuming 20 g/d, and progressively lower values for more distant consumption. Moreover, the ratio of monounsaturated to saturated fatty acids (MUFA:SFA) and the intake of *trans*-fatty acids (TRANS) was also directly standardised to *z*-scores. To obtain the total score for the MDP, the consumption of legumes, cereals, fruits, and vegetables and moderate consumption of alcohol and MUFA:SFA ratio are positively weighted (summed), for being considered beneficial components, while the consumption of *trans*-fatty acids, meat and dairy products are negatively weighted (subtracted), for being considered harmful components. Thus, higher scores configure higher adherence to MD assessed by the MDP, according to the following formula:
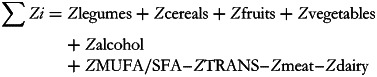
where Z*i* is the individual total standardised *z*-scores of nine components

Then, the total MDP score is converted into a percentage of adherence based on the sample variation range, as described in the following formula^(42)^:
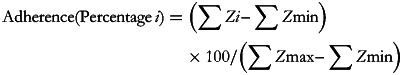
where *Z*min is the lowest *z*-score obtained in the sample; *Z*max is the highest *z*-score obtained in the sample.

Supplementary Figure 2 summarises the criteria adopted to construct the five selected dietary indexes, considering the method of calculation, dietary components, cut-off point, scoring system, scoring range, inclusion or not of non-Mediterranean foods and energy adjustment.

### Covariates

2015 ISA-Capital data were obtained through a structured questionnaire organised by thematic areas. In the present study, socio-demographic and anthropometric variables were included as follows: sex (male or female), age (20–59 and ≥60 years old), years of formal education (≤9, 10–12 or >12), per capita household income (≤1 minimum wage and >1 minimum wage), and self-reported weight (kg) and height (m), from which we calculated the body mass index (BMI; weight/height^2^) and classified as below or above normality range: ≥18⋅5 and <25 kg/m² for adults^(43)^ and >23 and <28 kg/m² for older adults^(44)^.

### Statistical analyses

The categorical variables were described as relative frequency (percentage), and continuous variables were presented as a mean and standard deviation. For the continuous variables, the type of distribution was investigated by the Kolmogorov–Smirnov test, histogram examination, and measures of skewness and kurtosis. Median and interquartile ranges (IQRs) were calculated to describe the scores for each dietary index presented by the study sample. The multicollinearity between multiple independent variables was tested in a multiple regression model. The variance inflation factor (VIF) was obtained to measure the amount of multicollinearity. Analyses considered the sampling weight for representativeness of the population of São Paulo.

The correlation between the total scores of the five calculated indexes (MDS, MedDietscore, MDP, MSDPS and MAI) was verified using Spearman's correlation test. A correlation coefficient ≥0⋅70 was considered a very strong correlation; from 0⋅40 to 0⋅69 as a strong correlation; from 0⋅30 to 0⋅39 as a moderate correlation; from 0⋅20 to 0⋅29, a weak correlation and from 0⋅00 to 0⋅19, no or negligible correlation^(45)^. In addition, considering the data metric as interval scales, after classifying the participants into tertiles (data driven using the Stata's command *xtile*) of adherence to the MD, linearly weighted Cohen's Kappa coefficient was estimated to measure the agreement between the indexes. Its interpretation followed the classification: fair concordance when the coefficient was ≤0⋅40; moderate concordance for coefficients from 0⋅41 to 0⋅60 and substantial or almost perfect concordance when the coefficient ranged from 0⋅61 to 0⋅80 and >0⋅80, respectively^(46)^.

Considering the differences between the indexes and the difficulty of identifying the most appropriate ones to assess the adherence to the MD, the convergent validity and model fit measures were assessed using confirmatory factor analysis (CFA). In this analysis, scores obtained in each food component of dietary indexes were adopted. The following goodness-of-fit (GOF) indices were applied: Comparative fit index (CFI >0⋅95), Tucker–Lewis Index (TLI > 0⋅95), Residual mean square error of approximation with 90% CI (RMSEA ≤ 0⋅06 and upper limit of the 90% CI < 0⋅08) and Standardised root mean square residual (SRMR ≤ 0⋅05)^(47)^. Robust maximum likelihood (MLR) and robust weighted least squares (WLSMV) estimators were used when observed variables were continuous and categorical, respectively. Factor loadings ≥0⋅50 were considered to identify relevant correlations between each model's observed variables (food components intake) and latent variables^(48)^. The internal consistency reliability of each model was also evaluated by calculating McDonald's omega with values equal to or higher than 0⋅7, which was considered acceptable^(49)^.

The statistical significance was set for all the variables at *P* < 0⋅05. The statistical package Stata v.14 (Stata Corp., LP, College Station, TX, USA) was used to perform the descriptive and comparative analysis, while the Mplus Version 8⋅7 (Los. Angeles, CA) to fit the CFA models and their reliability.

## Results

Regarding socio-demographic characteristics of the participants, 50⋅3% were women, 70⋅5% were between 20 and 59 years old, 41⋅6% had less than 9 years of education, and 59⋅7% had per capita household income higher than one Brazilian minimum wage. Also, 54⋅8% were classified as within the normality based on their BMI values (Table 1). The VIF values (all between 1⋅0 and 1⋅2) indicated no considerable collinearity in the regression model.

**Table 1.** Socio-demographic characteristics and nutritional status of the participants

Variable	*n*	%^a^	95% CI
Sex			
Male	554	49·7	46·2–53·2
Female	633	50·3	46·7–53·7
Age (years)			
20–59	643	70·5	67–73·8
≥60	544	29·5	26·2–33
Years of formal education			
≤9	605	41·6	37·9–45·3
10–12	320	30	27·2–33
>12	260	28·4	24·4–32·7
Per capita household income			
≤1 MW^b^	419	40·3	35·3–45·5
>1 MW^b^	582	59·7	54·5–64·7
BMI^c^ (kg/m²)			
Below normality range	118	7·1	5·6–8·8
Normality range	696	54·8	50·8–58·7
Overweight	347	38·1	34·3–42·1

a Analyses considered the sampling weight.

b Minimum wage.

c Body mass index (*Adults*: below normality range: <18·5 kg/m²; Normality range: 18·5–24·9 kg/m²; Excess body weight: ≥25 kg/m²; *Older adults*: below normality range: <23 kg/m²; Normality range: 23–27·9 kg/m²; Overweight: ≥28 kg/m²).

Table 2 shows each index's median, interquartile range, minimum and maximum values. The MDS and MedDietscore had median values centred on a moderate adherence to the MD, while low medians were found for the other three dietary indexes (MAI, MDP and MSDPS). The maximum score was achieved only to the MDP index since its final score is calculated in the percentage of adherence to the MD. This result was followed by the MedDietscore and MDS indexes presenting maximum scores (40 and 8, respectively) closest to their highest scoring range.

**Table 2.** Descriptive analysis of the Mediterranean dietary indexes

Indexes (scoring range)	Median	IQR	Min–Max
MedDietscore (0–55)	29	26–31	15–40
MDP (0–100)	27·5	20·7–35·1	1·9–100
MDS (0–9)	4	3–5	0–8
MAI (0–100)	0·5	0·4–0·7	0·1–3·5
MSDPS (0–100)	12·6	9·9–16	1·9–32·9

Analyses considered the sampling weight.

IQR, interquartile range; Min, minimum; Max, maximum; MedDietscore, Mediterranean diet score; MDP, Mediterranean dietary pattern; MDS, Mediterranean diet scale; MAI, Mediterranean Adequacy Index; MSDPS, Mediterranean-Style Dietary Pattern Score.

All the indexes were positively inter-correlated, although with different strengths. The two strongest correlations were found for the MDP with MAI (0·76, 95% CI 0·74–0·79) and with MDS (0·72, 95% CI 0·69–0·75). The MDS and MedDietscore were strongly inter-correlated (0·50, 95% CI 0·45–0·54) and both showed a strong correlation with the MAI index as well (0·62, 95% CI 0·58–0·65 and 0·50, 95% CI 0·46–0·55, respectively). The MSDPS index presented the weakest correlations, with MDS (0·26, 95% CI 0·21–0·32) and MDP (0·29, 95% CI 0·23–0·34) (Table 3).

**Table 3.** Correlation among indexes of adherence to the Mediterranean dietary pattern

	Correlation coefficient^a^ (95% CI)			
Indexes	MedDietscore	MAI	MDP	MSDPS
MDS	0·50 (0·45–0·54)^b^	0·62 (0·58–0·65)^b^	0·72 (0·69–0·75)^c^	0·26 (0·21–0·32)^d^
MedDietscore		0·50 (0·46–0·55)^b^	0·47 (0·43–0·52)^b^	0·34 (0·29–0·39)^e^
MAI			0·76 (0·74–0·79)^c^	0·33 (0·27–0·38)^e^
MDP				0·29 (0·23–0·34)^d^

95% CI, 95% confidence Interval; MedDietscore, Mediterranean diet score; MAI, Mediterranean Adequacy Index; MDP, Mediterranean dietary pattern; MSDPS, Mediterranean-Style Dietary Pattern Score; MDS, Mediterranean diet scale.

a Spearman correlation coefficient.

b Strong correlation (correlation coefficient from 0·4 to 0·69).

c Very strong correlation (correlation coefficient ≥0·70).

d Weak correlation (correlation coefficient from 0·2 to 0·29).

e Moderate correlation (correlation coefficient from 0·3 to 0·39).

Table 4 shows the agreement among the indexes in terms of tertiles (data driven) classification, which seems consistent with the correlation results. The highest percentage of agreement was found between MDP and MAI indexes (80·7%), with a kappa coefficient representing a moderate agreement (0·57). A moderate agreement was also observed between MDP and MDS indexes (78%). Although the other indexes showed above 70% of agreements (MDS *v.* MedDietscore and MDS *v.* MAI), the kappa coefficients pointed to the weaker agreement in these cases.

**Table 4.** Percentage agreement among the degree of adherence to the Mediterranean diet evaluated by the five indexes

Indexes	MedDietscore	MAI	MDP	MSDPS
MDS	71·9%	74·3%	78%	64·4%
	0·34^a^	0·40^a^	0·48^a^	0·16^a^
	<0·001	<0·001	<0·001	<0·001
MedDietscore	100%	69·1%	69·6%	66·2%
		0·32^a^	0·33^a^	0·25^a^
		<0·001	<0·001	<0·001
MAI		100%	80·7%	65%
			0·57^a^	0·21^a^
			<0·001	<0·001
MDP			100%	63·6%
				0·18^a^
				<0·001

MedDietscore, Mediterranean diet score; MAI, Mediterranean Adequacy Index; MDP, Mediterranean dietary pattern; MSDPS, Mediterranean-Style Dietary Pattern Score; MDS, Mediterranean diet scale.

a Linearly weighted Cohen's Kappa coefficient: fair concordance: ≤0·40; moderate concordance: 0·41 to 0·60; substantial concordance: 0·61–0·80; almost perfect >0·80.

**Fig. 1.**
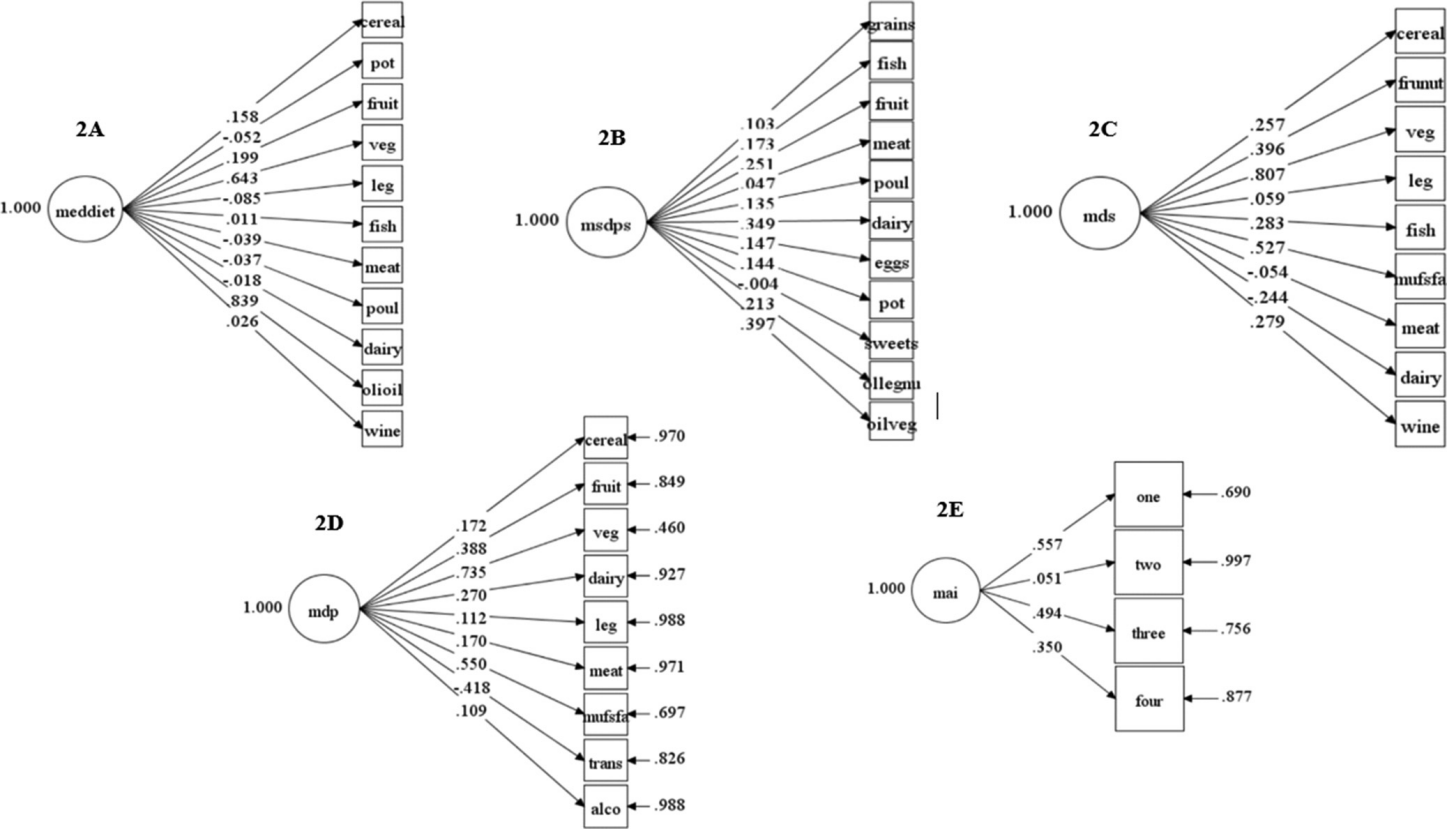
Confirmatory factor analysis for each Mediterranean dietary index. Circles: latent variables. Squares: observed variables. The straight arrows connect the observed and the latent variables: standardised factor loadings. The free straight arrows attached to observed variables indicate measurement errors. (a) MedDietscore’ construct: potato (pot), vegetables (veg), legumes (leg), poultry (poul), olive oil (olioil); (b) MSDPS’ construct: whole grains (grains), fruits, poultry (poul), dairy, potato (pot), olives, legumes and nuts (ollegnu), olive oil and vegetables (oilveg); (c) MDS’ construct: fruits and nuts (frunut), vegetables (veg), legumes (leg), MUFA:SFA (mufsfa); (d) MDP’ construct: vegetables (veg), legumes (leg), MUFA:SFA (mufsfa), *trans*-fatty (trans), alcohol (alco); (e) MAI’ construct: one (carbohydrate group), two (protective group), three (land animal group), four (sweets).

The final models interpreted in the CFA representing the MDS, MAI, MDP and MedDietscore consisted of their original structures. However, it was necessary to modify the MSDPS structure to get the model fit (final model diagrams in Fig 1).

As observed from the data described in Table 5, the MedDietscore (RMSEA = 0·033, 90% CI 0·02–0·042; SRMR = 0·042) and the MSDPS (RMSEA = 0·028, 90% CI 0·019–0·037; SRMR = 0·031) were the only indexes that had GOF indices (considering the RMSEA and SRMR values), indicating good models fit. All the models presented low values for incremental fit indices and significant *P*-values for the *χ*² test. Also, the McDonald's Omega coefficients for each index remained close to each other, with MDS having the highest value (0·50), still indicating unacceptable internal consistency.

**Table 5.** Results of confirmatory factor analysis for models of each dietary index

Dietary index	*χ*^2^	df	*P*	RMSEA (90% CI)	SRMR	CFI	TLI	Omega
MDP	196·483	27	<0·001	0·073 (0·063–0·082)	0·050	0·761	0·681	0·40
MedDietscore	102·094	44	<0·001	**0·033 (0·025–0·042)**	**0·042**	0·758	0·697	0·30
MAI	22·821	2	<0·001	0·094 (0·062–0·130)	**0·030**	0·853	0·559	0·40
MDS	86·688	27	<0·001	**0·043 (0·033–0·05)**	0·104	0·821	0·761	0·50
MSDPS	84·193	44	0·003	**0·028 (0·019–0·037)**	**0·031**	0·637	0·546	0·30

Results in bold meet recommended values.

*χ*², Chi-squared test; df, degrees of freedom; RMSEA, root mean square error of approximation; SRMR, standardised root mean square residual; CFI, comparative fit index; TLI, Tucker–Lewis Index; MDP, Mediterranean dietary pattern; MedDietscore, Mediterranean diet score; MAI, Mediterranean Adequacy Index; MDS, Mediterranean diet scale; MSDPS, Mediterranean-Style Dietary Pattern Score.

Regarding the food groups that make up the structure of each dietary index, the highest correlations were found for vegetables reflecting the construct of MedDietscore (factor loading = 0·64) (Fig. 1(a)), MDS (factor loading = 0·81) (Fig. 1(c)) and MDP (factor loading = 0·74) (Fig. 1(d)). Also, higher factor loadings were observed to olive oil reflecting the construct of MedDietscore (factor loading = 0·84) (Fig. 1(a)); MUFA:SFA ratio reflecting the construct of MDS (factor loading = 0·53) (Fig. 1(c)) and MDP (factor loading = 0·55) (Fig. 1(d)); cereals and legumes (as group one) reflecting the construct of MAI (factor loading = 0·56) (Fig. 1(e)). All these correlations were statistically significant.

## Discussion

The present study investigated the convergent validity of five indexes in determining the adherence to MD in a sample of Brazilian adults and older adults. Two indexes, the MedDietscore and MSDPS, showed the best convergent validity from CFA. The CFA also found higher factor loadings (≥0·50) for vegetables, olive oil, MUFA:SFA ratio and cereals with legumes among the different dietary indexes. In turn, the MDP showed the strongest correlation and highest agreement with the other indexes, followed by MAI and MDS. The MDP and MDS encompass nutrients, food group indicators and energy-adjusted cut-off points, while MAI differs in all these aspects. However, despite not including non-Mediterranean foods, the MDP includes in the nutrient list the food content of *trans*-fatty acids, and MAI considers industrialised processed foods in the list, which are considered sources of *trans*-fatty acids. Also, the MDP is the only index that uses standardised consumption (*z*-score).

The MDS, one of Greece's most widely used indexes, presented the best performance in the correlation and agreement analysis but not in the CFA analysis. In turn, the MedDietscore had more recommended criteria in the structure of an index, like cut-off points based on recommendations and more detailed scoring ranges (broader and more subdivided scores)^(21)^. Compared with our data, Aoun *et al.*^(50)^ studied 100 healthy Lebanese adults and found a lower percentage of agreement (65%) between MDS and MedDietscore indexes and a higher kappa coefficient (*r* = 0·69; *P* < 0·001, which indicated moderate agreement); the authors categorised the scores into low and high adherence based on the 50th percentile instead of tertiles.

Another study carried out with undergraduate students from Spain (*n =* 324) compared the adherence to the MD using ten different indexes; the three higher correlations were found between MDP *v*. MAI (0·82); MAI *v*. MSDPS (0·80) and MDS *v*. relative Mediterranean diet, or rMED (0·77). A good correlation with rMED was expected because it consists of a Spanish variation of the MDS^(36,51)^. Unlike our correlation results, the MSDPS had the cut-off points of the dietary guideline for the Greek population, which probably contributed to this higher correlation. The authors also found that MDS correlated more strongly with MAI (0·6) and MDP (0·6) compared with MedDietscore (0·3 and 0·34, respectively)^(51)^.

Milà-Villarroel *et al.*^(51)^ used exploratory factor analysis to identify which of the ten indexes defined the same factor; this analysis was interpreted as adherence to the MD. However, different from our analysis, the authors adopted the final score of each index instead of using the food components as observed variables. They found higher factor loadings for MDS, MAI and MDP and lower factor loadings for MSDPS and MedDietscore. These findings reinforce that the indexes using Mediterranean recommendations as cut-off points set the findings apart from others when applied in non-Mediterranean populations. Another important interpretation is using an index adapted for another Western population, like the MSDPS. It included the so-called western foods, which does not necessarily make this choice the best for our sample; we observed low median and maximum values for this index. Also, despite the good values of the GOF indices, RMSEA and SRMR, for the MSDPS in our study, we needed to modify the original version to make it possible to estimate its model fit.

Although the absolute fit indices adopted, RMSEA and SRMR, suggested a good fit^(47)^ for the models representing the MedDietscore and MSDPS, they had low values for the Incremental fit indices, CFI and TLI^(47)^. As the Incremental fit indices compare adjustment quality between the hypothetical model and the null model^(52–54)^, the measured variables that made up each of the two models might be poorly correlated. Indeed, this was expected since the observed variables represent a dietary pattern that, considering its concept, people adhere to more or less^(55)^.

As recommended to determine convergent validity, we analysed further aspects of the CFA, like the factor loadings^(56)^. Even in the models with the best fit indices, none of the dietary indexes presented factor loadings ≥0·50 for all the observed variables to meet the convergent validity criterion^(48)^. Interestingly, vegetables, olive oil and MUFA:SFA (observed variables) had similar factor loadings (≥0·50) in different models. These findings were similar to those of Milà-Villarroel *et al.*^(51)^, showing that there seems to be a consensus on foods that cannot be disregarded in the Mediterranean diet.

The low factor loadings of foods such as fish and wine were probably due to the low consumption by our population. In Brazil, industrialised foods are part of the population's eating habits. Foods such as olive oil, wine, fish and oilseeds are less frequently consumed in some regions of the country, such as in the Southeast^(57,58)^, making studying this topic challenging. Still, a greater consumption of these foods, even in Mediterranean countries, is also challenging because they are less affordable to most population groups^(59)^. From our results, investigating the consumption of these foods in combination with other components, such as fruits and oilseeds, which are the same component of the MDS, could be a good strategy. Also, when combining vegetables with olive oil in the MSDPS, the model fit improved.

It is important to highlight some limitations of our work. We collected the 24HDR, which is prone to measurement error, such as memory bias and underreporting certain foods. Also, considering the sizeable territorial extension of Brazil and the different cultural food habits among the regions of the country, these cross-sectional data analyses are not nationally representative and do not reflect rural communities within Brazil, but rather urban dwelling Brazilian civilians who reside in Sao Paulo, SP, Brazil. Although not all convergent validity points were met, it is essential to consider the good results obtained by the RMSEA since it has been considered one of the most relevant fit indices.^(60)^ Also, we found, from CFA analysis, high measurement errors for most of the results, but further aspects of this analysis were considered when interpreting the results.

On the other hand, as strong points, our results brought substantial evidence when evaluating the adherence to the MD in a non-Mediterranean population. Our study is the first to test the convergent validity using CFA of different MD indexes in adults and older adults from São Paulo. As far as we know, no previous studies performed this same analysis on any other Latin-American population. The calculation of the five indexes was performed considering usual dietary data by removing within-person variation from two 24HDR. Food consumption was obtained following a carefully developed protocol to minimise bias. The CFA brought results that can guide researchers in choosing an existent index to be applied to the Brazilian population. Importantly, these results also revealed relevant food components, the metric, and scoring of the index for further studies interested in adapting a dietary index based on the Mediterranean diet for the Brazilian population, considering the absence of a specific index for the Brazilian setting.

In conclusion, the five dietary indexes classified the population similarly, but not necessarily regarding adherence to the MD. The MDS, MAI and MDP were more correlated and had a higher agreement. Although they were proposed to achieve the same objectives, only the MSDPS and the MedDietscore performed better in evaluating the adherence to the MD in our sample. The MedDietscore excelled regarding this last aspect since it was evaluated based on its structure and original proposal. Also, olive oil, vegetables, MUFA:SFA ratio and cereals with legumes were the components most relevant to characterise this dietary pattern in the present study. Our findings showed important aspects of similarities and differences between the Mediterranean dietary indexes. These results constitute an important basis for future studies intending to investigate the effects of following the Mediterranean diet on different health outcomes in non-Mediterranean populations.

## Supplementary material

For supplementary material accompanying this paper visit https://doi.org/10.1017/jns.2022.123. click here to view supplementary material
