# Supplementary material for: Comparison and convergent validity of five Mediterranean dietary indexes applied to Brazilian adults and older adults: data from a population-based study (2015 ISA-Nutrition)
Source: J Nutr Sci. 2023 Jan 26;12:e12. doi: 10.1017/jns.2022.123 (PMC9947597; doi:10.1017/jns.2022.123)
Supplement: Supplementary file 1 [file S2048679022001239sup001.docx]

**Supplementary material**

**Supplementary Table 1.** Description of the dietary indexes excluded, and the criteria applied for the exclusion.

| **Authors (year)** | **Dietary index** | **Country** | **Scoring range** | **Reason for exclusion** |
| --- | --- | --- | --- | --- |
| Sotos-Prieto et al.^24^ | MEDLIFE^a^ | Spain | 0-28 | Structure questionnaire/not applicable to 24HDR data |
| Gerber et al.^25^ | Med-DQI-2000^b^ | South of France | 0-14 | MedDietScore variation |
| Gerber et al.^25^ | Med-DQI-f-2000 | South of France | 0-16 | MedDietScore variation |
| Gerber^26^ | Med-DQI-2006 | France | 0-14 | MedDietScore variation |
| Agnoli et al.^27^ | IMI-2011^c^ | Italy | 0-11 | MDS variation |
| Knoops et al.^28^ | MAI-2006^d^ | Mediterranean and non-Mediterranean European countries | Not normalized | Not normalized |
| Schröder et al.^29^ | MEDAS-2011^e^ | Spain | 0-14 | Structure questionnaire/not applicable to 24HDR data |
| Domınguez et al.^30^ | MEDAS-2013 | Spain | 0-13 | Structure questionnaire/not applicable to 24HDR data |
| Haveman-Nies et al.^10^ | MDS-2001^f^ | Western countries (USA, Europe) | 0-8 | MDS variation |
| Haveman-Nies et al.^31^ | MDS-2002 | Western countries (Europe) | 0-7 | MDS variation |
| Knoops et al.^32^ | MDS-2004 | Western countries (Europe) | 0-8 | MDS variation |
| Cade et al.^33^ | MDS-2011 | United Kingdom | 0-10 | MDS variation |
| Trichopoulou et al.^34^ | mMDS^g^ | Mediterranean and non-Mediterranean European countries | 0-9 | MDS variation |
| Yang et al.^11^ | mMDS-2014 | USA | 0-42 | Structure questionnaire/not applicable to 24HDR data |
| Fung et al.^12^ | aMED-2005^h^ | USA | 0-9 | MDS variation |
| Sanchez-Taınta et al.^35^ | MeDiet-2008^i^ | Spain | 0-14 | Structure questionnaire/not applicable to 24HDR data |
| Goulet et al.^36^ | MS-2003^j^ | Canada | 0-44 | MedDietScore variation |
| Buckland et al.^37^ | rMED-2009^k^ | Spain | 0-18 | MDS variation |
| Buckland et al.^38^ | arMED-2013^l^ | Mediterranean and non-Mediterranean European countries | 0-16 | MDS variation |

^a^Mediterranean Lifestyle index. ^b^Mediterranean Diet Quality Index. ^c^Italian Mediterranean Index. ^d^Mediterranean Adequacy Index. ^e^Mediterranean Adherence Diet Screener. ^f^Mediterranean Diet Score. ^g^Modified Mediterranean Diet Score. ^h^Alternate Mediterranean Diet Score. ^i^Mediterranean Food Pattern. ^j^Mediterranean Score. ^k^Relative Mediterranean Diet. ^l^Adapted Relative Mediterranean Diet


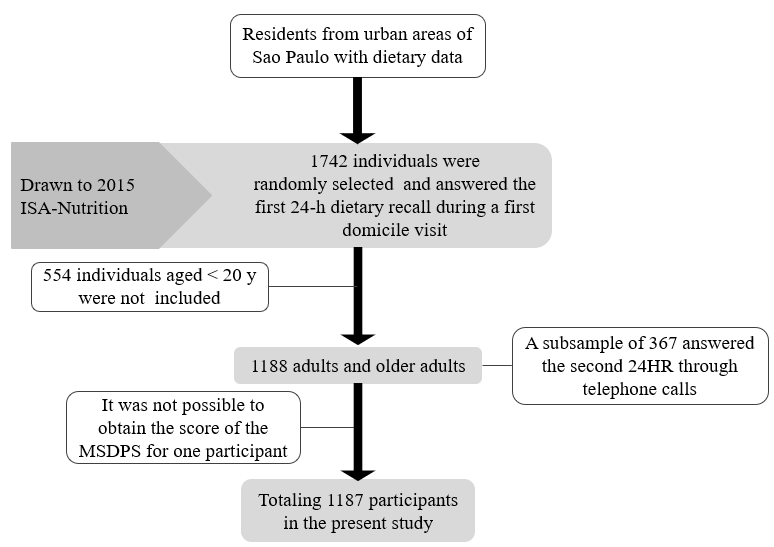


**Supplementary Figure 1**. Description of the sample in the 2015 Health Survey of São Paulo with Focus on Nutrition Study (2015 ISA-Nutrition) included in the present study.


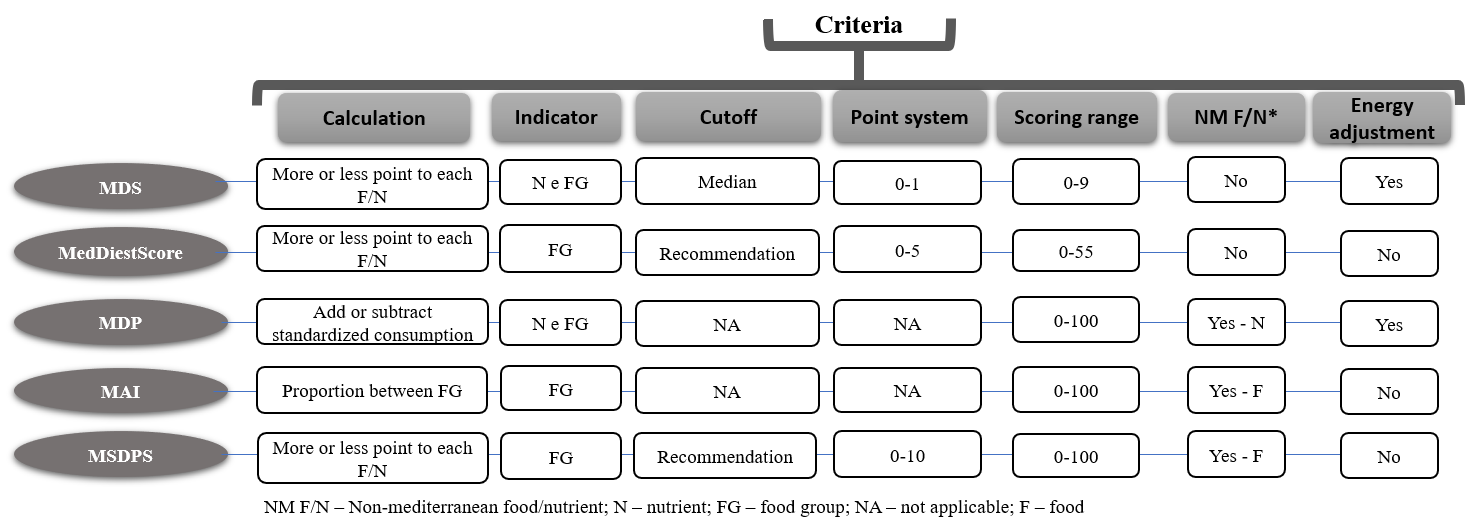


**Supplementary Figure 2**. Criteria adopted in constructing the five selected dietary indexes.
